# An Approach for Studying the Direct Effects of Shock Waves on Neuronal Cell Structure and Function

**DOI:** 10.3390/cells14080563

**Published:** 2025-04-09

**Authors:** Michael Hanna, Bryan J. Pfister

**Affiliations:** 1 Biomedical Engineering Department, Tandon School of Engineering, New York University, Brooklyn, NY 10012, USA; mh7376@nyu.edu; 2 Center for Injury Biomechanics, Materials and Medicine, Department of Biomedical Engineering, New Jersey Institute of Technology, Newark, NJ 07102, USA

**Keywords:** traumatic brain injury, blast injury, in vitro model, intracranial pressure

## Abstract

Recent U.S. military conflicts have underscored the knowledge gap regarding the neurological changes associated with blast-induced traumatic brain injury (bTBI). In vitro models of TBIs have the advantage of following the neuronal response to biomechanical perturbations in real-time, which can be exceedingly difficult in animal models. Here, we sought to develop an in vitro approach with controlled blast biomechanics to study the direct effects of the primary shock wave at the neuronal level. A blast injury apparatus mimicking the human skull and cerebrospinal fluid was developed. Primary neuronal cells were cultured inside the apparatus and exposed to a 70 kPa peak blast overpressure using helium gas in a blast tube. Neuronal viability was measured 24 h after blast exposure. The transmission of the pressure wave through the skull is believed to be a factor in injury to the cells of the brain. Three thicknesses in the apparatus wall were studied to represent the range of thicknesses in a human skull. To study the transmission of the shock wave to the neurons, the incident pressure at the apparatus location, as well as internal apparatus pressure, were measured. Analysis of the internal pressure wave revealed that wave oscillation frequency, not amplitude, was a significant factor in cell viability after a bTBI. This finding is related to the viscoelastic properties of the brain and suggests that the transmission of the shock wave through the skull is an important variable in blast injury.

## 1. Introduction

Recent U.S. military conflicts have underscored the knowledge gap regarding blast-induced traumatic brain injury (bTBI). Since 2001, 60–80% of all U.S. service member casualties in Iraq and Afghanistan have resulted from improvised explosive devices [1,2]. In the traumatic brain injury (TBI) field, it is well accepted that blunt head injury causes rapid deformation of the brain tissue and neuronal cells, leading to calcium dysregulation, cytoskeletal degradation, and axonal degeneration [3,4,5,6,7,8,9,10,11]. The initiating TBI event is a dynamic mechanical loading (blunt, inertial, and blast) to the head that can differ significantly in terms of speed, direction, and duration [12,13,14]. Indeed, the initiation of TBI from a blast is different than blunt forms of head injury, and the mechanical mechanism leading to injury is much less understood. In the case of bTBI, how the mechanical perturbation induces injury is unknown.

Currently, there is a significant effort focused on animal TBI models of blast exposure [15,16]. In vitro models of TBI, however, have the advantage of following the neuronal response to biomechanical perturbations in real-time, which can be exceeding difficult in animal models [17]. How rapid dynamic mechanical deformation of the neuron leads to injury progression has been best described in reduced in vitro models [18,19,20,21,22]. A limitation of in vitro models is the absence of the structural organization of all the cells and tissues of the brain. However, the simplification of a neuronal tissue culture model allows the study of the direct effect of the shock wave on neuronal injury. Here, we engineered an in vitro approach with controlled blast biomechanics to study the direct effects of the primary shock wave at the neuronal level.

A limited effort of in vitro blast modeling has been performed using a few different sources to create the shock wave [17,22,23,24,25,26,27,28]. The results from these models have not revealed a significant change in cell viability at exposure levels comparable to human exposure, however, changes in intracellular sodium ions, calcium ions, cell permeability, and reactive oxygen species have been reported [2,29,30]. In these models, the designs are such that the shock wave is attenuated through a large water barrier or the applied blast wave has a very short duration of less than 1 ms [25,29]. One model simulated human anatomy by enclosing cell cultures in a plastic cover to represent the human skull [28]. To better understand the effects of the primary blast, an in vitro model needs to be designed such that the neurons are exposed to similar shock waveforms within the structure of a human head. Studies indicate that the skull and cerebral spinal fluid (CSF) can have a large effect on the transmission of the shock wave in the brain [31,32]. Our goal was to consider a model that includes the features of the skull and CSF to more closely replicate the effects from the direct action of a shock wave on the cells of the brain.

Shock wave transmission refers to the movement of the shock wave from one medium to another. This is a complex physics phenomenon and can be empirically studied by considering the incident shock waveform at the outer surface of any sample (i.e., the surface of the skull) and the internal, transmitted waveform inside the sample at the region of interest (i.e., the intracranial space) [33,34,35]. In this study, we created a model that could be exposed to a Friedlander shock wave using a compressed gas shock tube with adjustable parameters for the thickness of the skull and CSF [36,37,38].

## 2. Methods

Inspired by previously published research on surrogate head models, our preliminary experiments started by culturing neurons inside a full-size surrogate head model and then exposing them to a shock wave [39,40]. However, a full replicate of the human head presented significant challenges and confounding experimental variables for a reduced model, including a large physical size and weight, variations in neuronal sample placement, and shock wave exposure. In this study, we designed a simplified enclosure that allows precise shock wave exposure to a primary neuronal culture. A round cylindrical enclosure symmetrically holds a sample amenable to cell culture methodology, a watertight cover that can be adjusted to varying skull material properties and thicknesses, and a surrounding media simulating cerebrospinal fluid.

### 2.1. In Vitro Blast Injury Apparatus

One consideration with existing in vitro models is that they orient cells parallel to the shock wave propagation where cells in the front would receive a higher pressure than the cells in the back [23,30]. To guarantee that all cells receive the same blast intensity, our design ensured the cells were on a plane normal to the direction of blast wave motion. In addition, our initial experiments found that the use of tissue culture plastic led to 100% cell detachment after the blast. We found that a compliant silicone-based cell culture substrate resolved these issues.

**Figure 1:**
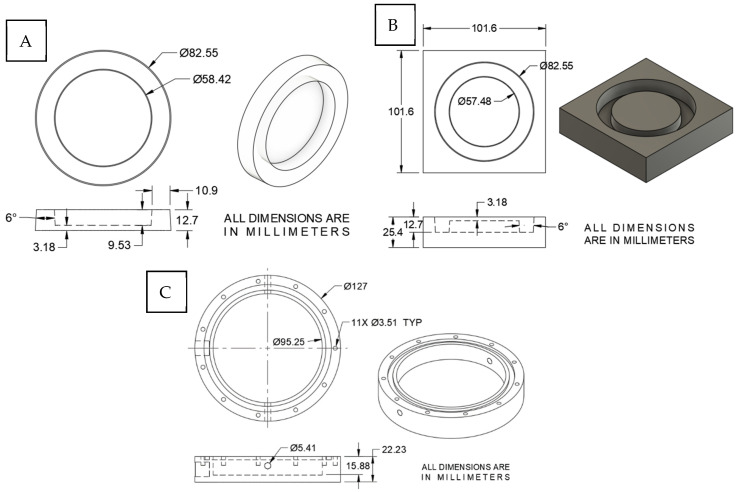
Cell blast apparatus drawings. (**A**) PDMS cell culture substrate drawings. (**B**) The mold for the PDMS culture wells. (**C**) The outer cylinder of the cell blast apparatus.

To develop a soft cell culture substrate for the blast apparatus, we used polydimethylsiloxane (PDMS) to create custom culture wells. This choice was guided by our previous experience in culturing neurons on PDMS in neuronal stretch injury systems [41,42]. PDMS has many advantages that are relevant to our application. PDMS is simple to process, biocompatible, and transparent in microscopy [43]. The PDMS cell culture substrate was designed with a plating area (2680 mm^2^) accommodating about 2 million cells per sample, which is adequate for western blotting. The bottom thickness of the PDMS cell culture substrate was limited to 3 mm to allow for sufficient microscopic examination. Since the design did not provide mechanical stability for handling and insertion into the blast apparatus, 10 mm thick supporting sides were formed surrounding the plating area, as shown in Figure 1. The PDMS mold was drawn in Fusion 360 (v16.11.1 by Autodesk San Francisco, CA, USA), converted into CNC machining code, and machined from a 101 mm square block of aluminum on a CNC mill TM-1 (HAAS Oxnard, CA, USA). The mold was polished using diamond lapping paste to achieve the best optical clarity in the PDMS. The polishing was done in 6 steps, starting with a 12-micron diamond paste then 8, 4, 2, 1, and 0.5 microns, respectively.

**Figure 2:**
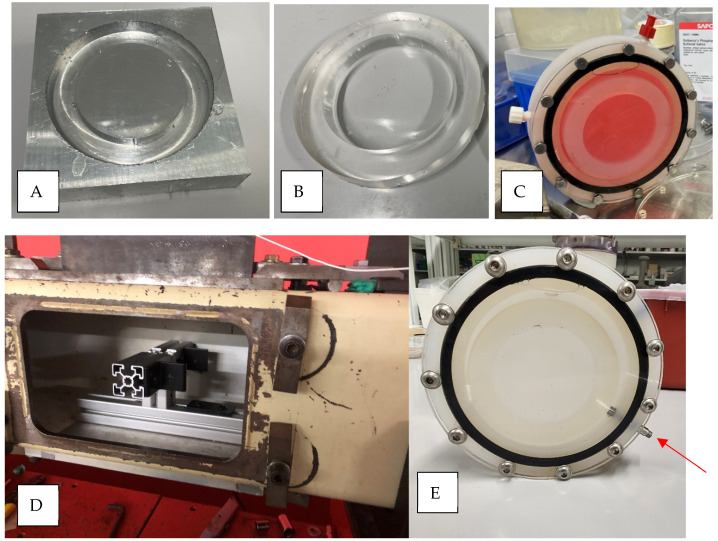
The cell blast apparatus. (**A**) The aluminum mold for casting the PDMS culture wells. (**B**) The cast of the PDMS culture well. (**C**) The fully assembled blast injury apparatus before mounting in the blast tube. (**D**) Blast tube mounting fixture for the injury apparatus. (**E**) Injury apparatus with a pressure sensor.

To cast the PDMS culture well, a PDMS kit (Sylgard 184, Dow Corning, Midland, MI, USA) was prepared by mixing the base and curing agent in a ratio of 10 parts base to 1 part curing agent by weight, agitated to remove air bubbles, poured into the mold, and cured for 45 min at 75 °C. The PDMS was removed from the mold, washed with soap and water, dried, plasma cleaned for 3 min, and autoclaved prior to plating cells. The final product of the PDMS culture wells, as well as the aluminum mold, are shown in Figure 1 and Figure 2.

The blast injury apparatus consists of a custom-machined cylindrical container made from autoclavable polypropylene and a clear acrylic cover to house the neuronal culture within the shock tube, as shown in Figure 2. The polypropylene cylinder was fabricated to fit the PDMS cell culture substrate and a rubber O-ring to liquid seal the apparatus. Two Luer lock openings on the side allow for injecting media into the sealed container and releasing air bubbles. To understand the importance of shock wave transmission through a skull, we tested three different cover thicknesses (1/8″ [3.125 mm], 3/16″ [4.8 mm], and 1/4″ [6.35 mm]), each within the published range of the human skull (2–16 mm) [44,45,46,47]. In addition, Young’s modulus of acrylic is 2.76–3.00 GPa, which is within the range of the human cranial bone tensile modulus of 0.45–10.0 GPa [47,48,49,50,51]. Cell culture media is a good simulant of cerebrospinal fluid, both with viscosity comparable to water [52,53]. The cylinder was designed in Autodesk Fusion 360 and machined with a similar procedure as explained before for the mold, as shown in Figure 1C.

### 2.2. Primary Neuronal Culture

All procedures followed the guidelines established in the Guide for the Care and Use of Laboratory Animals and were approved by the Rutgers University Institutional Animal Care and Use Committee. The pregnant Sprague Dawley rats were obtained from Charles River Laboratories (Fairfield, NJ, USA), then anesthetized with CO_2_ and euthanized using CO_2_ after embryo extraction. A total of 12 rats were used in this study and 6 embryos from each rat were used in each isolation. The rat brain cortices were isolated from the E17 Sprague-Dawley rat embryos and stored in ice-cold HBSS containing Ca^2+^ and Mg^2+^. After rinsing with Ca^2+^ free and Mg^2+^ free HBSS, the cortices were mechanically disrupted by pipetting up and down (3–5 times). The media containing the cells was filtered with a nylon mesh filter with 100 μm pores, then a filter with 40 μm pores to remove large pieces of tissue. The remaining cortical cells were cultured on a poly-L-lysine (0.14 mg/mL)-coated custom-made polydimethylsiloxane [PDMS] well (2,000,000 cells per well in 57-mm) in NeuroBasal media containing 2% B-27 (Thermo Fisher Scientific, Gibco, Waltham, MA, USA), 1% penicillin-streptomycin, and 0.4 mM L-glutamine at 37 °C in a CO_2_ (5%) incubator. Four wells per isolation were used for each experimental group. All methods here are reported according to ARRIVE guidelines.

### 2.3. Shock Wave Exposure

Before adding the cultured cells and media into the blast injury apparatus, the cylindrical container was autoclaved, and the acrylic cover was sterilized using 70% ethanol. Inside a biological safety cabinet, the PDMS neuronal cultures (day 10 in vitro) were placed inside the cylindrical container, and the clear acrylic cover was then bolted to the container. Last, warm culture media (37 °C) was injected into the apparatus through the Luer lock connections (Figure 2C).

A compressed gas shock tube was used to replicate a free-field blast wave characterized by the Friedlander function [36,38,54]. The assembled blast apparatus with the neuronal culture was quickly mounted inside the tube, facing the blast wave, and blasted at a 70 kPa peak blast overpressure using helium gas, as shown in Figure 2. Blast TBI research commonly reports the incident pressure or the shock wave measured in the wall of the shock tube at the sample location. The brain tissue, however, is exposed to a transmitted shock wave after it passes through the facia and the skull of the head. The transmitted pressure wave can be referred to as the intracranial pressure wave, measured with a pressure sensor within the skull (cranium). In this study, the response to neurons was related to the internal pressure wave within the cell culture apparatus. The incident pressures were measured using high-frequency Tourmaline pressure transducers, model 134A24 (1000 psi maximum pressure, resonant frequency ≥ 1500 kHz, 0.2 µs rise time, PCB Piezotronics, Depew, NY, USA). A series of pressure sensors were distributed along the length of the shock tube to measure pressure–time profiles, including the incident shock wave at the location of the in vitro culture, as shown in Figure 5. To measure internal pressure (e.g., intracranial pressure), a pressure transducer model number 113B28 (PCB Piezotronics, Depew, NY, USA) was mounted through the side wall of the cylindrical housing and was in contact with the medium surrounding the PDMS culture well, as shown in Figure 2 (red arrow). This allowed for the measurement of the transmitted pressure wave from the incident shock wave, representative of the pressure profile presented to the neuronal cells. All data were recorded at a 1.0 MHz sampling frequency, and the typical acquisition time ranged from 50 to 200 ms [36]. After the blast, the apparatus was then disassembled under the sterile hood, the media was changed, and the PDMS culture wells with the cells returned to the incubator for further examination.

### 2.4. Pressure Wave Analysis

All data were analyzed using MATLAB version 9.14.0.2337262 (R2023a) Update 5 (Mathworks Natick, MA, USA); the Fourier transform for the pressure wave signal was calculated using MATLAB’s *fft* function, the mean pressure using the *mean* function, and the pressure impulse using the *trapz* function. The average pressure wave amplitude, which is the arithmetic average of the absolute values of the deviations of the peak pressure from the mean pressure, was calculated using the following equations:

$$WA=\frac{1}{N}\sum_{i=1}^{N}\left|x_{i}-\mu \right|\;$$

$$\mu =\frac{1}{N}\sum_{i=1}^{N}x_{i}$$

where *WA* is the average pressure wave amplitude.

The incident blast pressure profiles from the experiments were analyzed. The mean maximum pressure was 15.79 psi ± 3.74, the mean duration was 4.16 ms ± 0.64, and the mean impulse was 17.82 psi·ms ± 3.57. Based on the measured mean incident blast wave duration of 4.16 ms, we limited our analysis of the apparatus pressure (intracranial pressure) to 4 ms following the start of the blast overpressure.

### 2.5. Cell Viability

Using a colloidal dye assay, we assessed cell viability by the cells’ ability to exclude trypan blue. The cells were incubated in a 0.4% trypan blue solution for 30 min and then washed and counted using bright-field optics. The dead cells were distinguishable by their dark blue staining. The cells were injured on day 10 in vitro and viability counts were done 24 h after injury.

### 2.6. Statistics

All the experiments included a minimum of three technical replicates and each technical replicate had 4 wells each. Therefore, each experimental group had (n = 12), and 3 replicates with 4 wells each. All results are expressed as the mean ± standard deviation. One-way ANOVAs were used to determine the differences between the control and experimental conditions, and a probability value of *p* < 0.05 was considered significant.

## 3. Results

### 3.1. Cell Viability After Blast

We hypothesized that the magnitude of the shock wave would injure neurons in a dose-dependent manner. Indeed, the transmission of a shock wave to neuronal cells can be affected by the thickness of the skull [33,55]. Here, three blast apparatus cover thicknesses of 0.125″ (3.125 mm), 0.1875″ (4.8 mm), and 0.250″ (6.35 mm) were used to test injury to the neurons. The cell viability of the neuronal culture was measured from uninjured shams and injured groups (n = 12 per group), including four wells from three different cell isolations to remove potential bias. The uninjured sham had the same handling as the injured group, except that the cells were not subjected to a shock wave.

**Figure 3:**
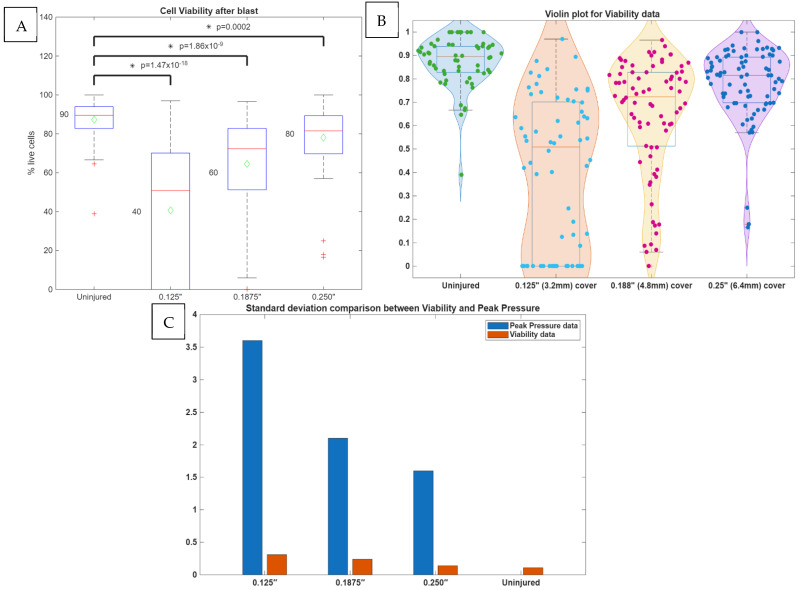
(**A**) ANOVA for cell viability after bTBI. (**B**) Violin plot of cell viability data after bTBI. (**C**) Standard deviation comparison between viability after bTBI and peak pressure.

**Figure 4:**
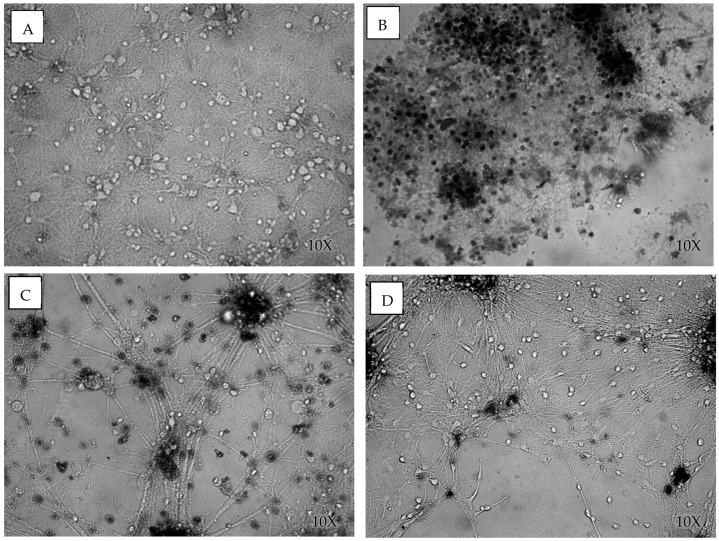
Transmission of the shockwave through the material thicknesses affects cell viability after blast exposure. Trypan blue staining was used to measure cell viability of the neuronal cultures exposed to the blast in (**A**) uninjured sham cultures, (**B**) injured cultures with a 1/8″ cover, (**C**) injured cultures with a 3/16″ cover, and (**D**) injured cultures with a 1/4″ cover.

The cover thickness had a large effect on cell viability following blast exposure. The control cultures had a viability of 0.87 ± 0.11, while the cells blasted with a cover thickness of 1/8″ (3.125 mm) had a viability of 0.41 ± 0.32 (F(120) = 106.51, *p* < 0.05), those blasted with a cover thickness of 3/16″ (4.8 mm) had a viability of 0.65 ± 0.24 (F(134) = 40.53, *p* < 0.05), and those blasted with a cover thickness of 1/4″ (6.35 mm) had a viability of 0.79 ± 0.14 (F(138) = 13.92, *p* < 0.05) (Figure 3 and Figure 4). As expected, the thicker cover (analogous to skull thickness) attenuated the immediate shock wave damage to the neurons. It is significant to point out that the data variability, presented by the standard deviation, decreased as the cover thickness increased, as shown in Figure 3C. The same pattern was observed also in peak pressure variability. The examination of the viability data through the violin plots (Figure 3B) shows the variability pattern. It is noticed that for the 1/8″ cover thickness, 30.15% of the data had 0% viability, and the rest of the data is normally distributed around a 58% average. Thus, the overall average of the data is 41%. For the 3/16″ cover thickness, the data is suggestive of bimodal distribution, where 30.38% of the data is normally distributed around viability of 35%, and the rest of the data has a mean of 77% viability, making the overall average of the data 65%. The 1/4″ cover thickness seems to be a single population, with an average viability of 78%. Bimodality in the 1/8″ cover as well as in the 3/16″ cover is reflected in higher standard deviations than in the 1/4″ cover. Therefore, cover thickness affects cell viability, as well as the variability in neuronal survival.

### 3.2. Analysis of Shock Wave Transmission

**Figure 5:**
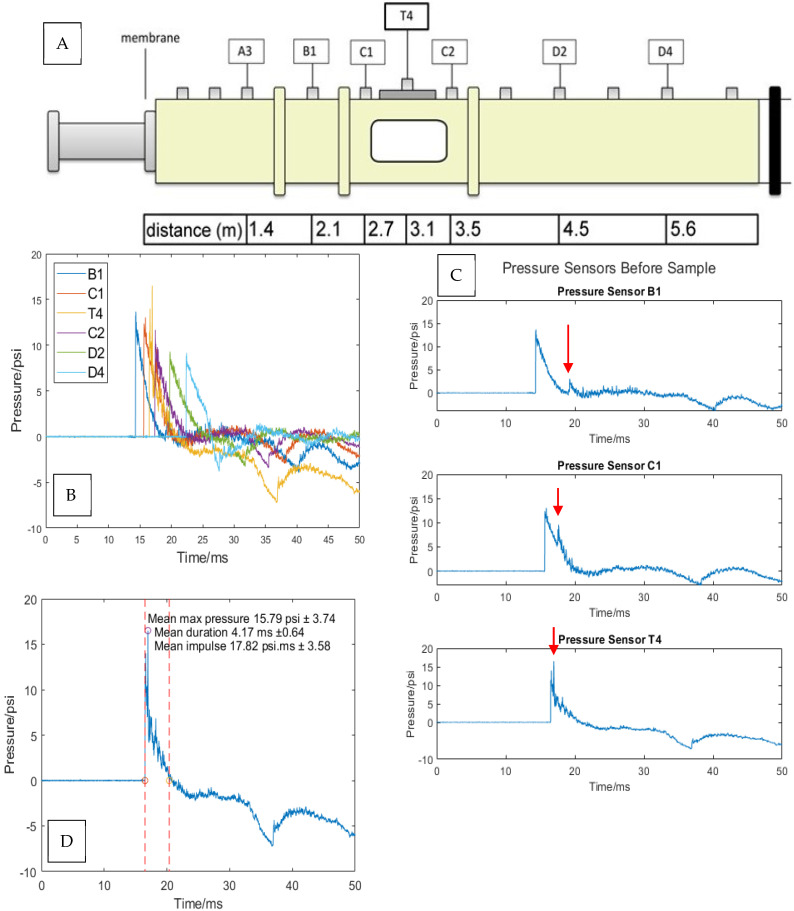
Blast tube pressure profiles. (**A**) A schematic of the shock tube. (**B**) The pressure profiles are measured at positions B1, C1, T4, C2, D2, and D4 along the blast tube from a single blast experiment. (**C**) The reflected wave detected in sensors B1, C1, and T4 was with a red arrow. (**D**) An analysis of the blast wave’s maximum pressure, duration, and impulse. The dotted lines represent the blast was duration from start to finish.

To understand the link between external blast pressure exposure and cell viability, we examined the pressure profiles inside the blast tube (incident pressure), as well as the transmitted pressure inside the blast apparatus (intracranial pressure). While most research studies report on the incident pressure, intracranial pressure is the transmitted shock wave and the direct mechanical insult subjected to the neurons. The intracranial pressure was measured using a pressure sensor installed inside the blast apparatus, as shown in the image in Figure 2E. Our blast tube allows pressure measurement at 6 different positions along the length of the tube [36]. Figure 5 illustrates the pressure measurement positions and the distance between them. The cell blast apparatus was placed right under pressure sensor T4. After carefully examining the pressure profiles before the cell blast apparatus (B1, C1, and T4), we noticed a reflected wave demonstrated by a second peak in the pressure profile (see red arrow in Figure 4B). The reflected wave was likely caused by the large surface area of the blast apparatus compared with the blast tube cross-sectional area. The effect of the reflected wave on our model will be discussed later when analyzing the apparatus pressure wave.

The blast tube pressure wave was analyzed, and the maximum pressure, duration, and impulse were calculated. The maximum pressure was reported as the largest recorded pressure during the blast event. The duration was calculated as the distance between two dotted red lines drawn at the point where the pressure signal crosses the *x*-axis, as shown in Figure 5D. The impulse is the area under the graph between the two dotted lines. The mean maximum pressure was 15.79 psi ± 3.74, the mean duration was 4.16 ms ± 0.64, and the mean impulse was 17.82 psi·ms ± 3.57 (n = 21 shock waves).

**Figure 6:**
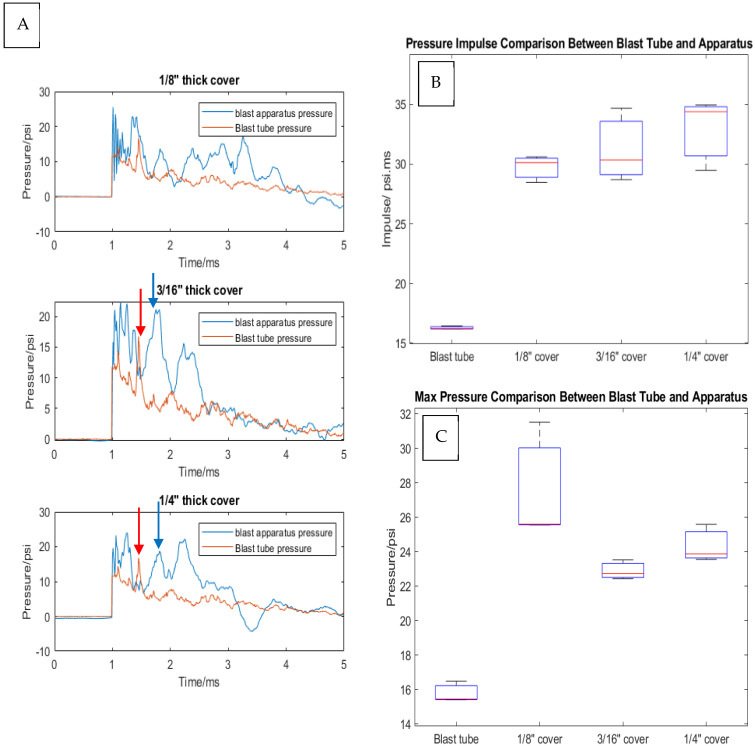
Comparison between the blast tube pressure and apparatus pressure. (**A**) A pressure plot of the blast tube pressure profile and apparatus pressure from a single experiment. Blue arrow represents the pressure spike inside the blast apparatus after the reflected wave shown in red arrow (**B**) A Box and Whiskers plot of the pressure impulse. (**C**) A Box and Whiskers plot of the maximum pressure. Notice the lagging peak, labeled with the blue arrow, that follows the reflected wave, labeled with the red arrow.

While the incident shock wave is the defined mechanical perturbation to the in vitro model, it is essential to understand how the blast wave is transmitted through the apparatus cover into the culture medium and the neuronal cells. This is analogous to the transmission of a shock wave through the skull and CSF to the brain (intracranial pressure). The internal apparatus pressure wave had 1.3 to 1.9 times higher peak pressure, and the pressure impulse was 1.6 to 2.1 higher in the apparatus pressure compared with the incident pressure (Figure 6B,C). In addition, careful examination of the pressure profiles inside the apparatus and the blast tube suggests that the reflected wave in the blast tube translated into a pressure peak inside the apparatus. Notice the lagging peak, labeled with the blue arrow in Figure 6A, that follows the reflected wave, labeled with the red arrow.

**Figure 7:**
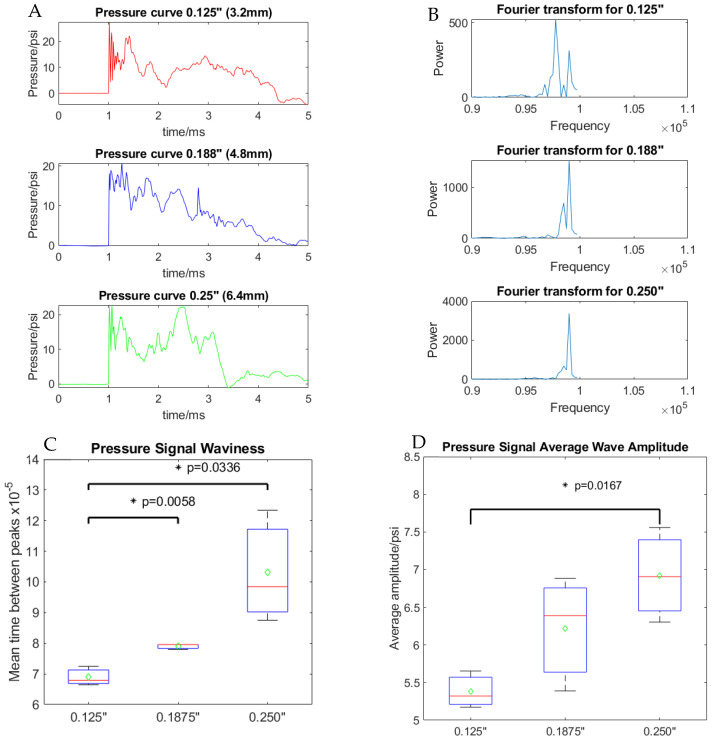
Pressure wave analysis. (**A**) Pressure profiles inside the injury apparatus (intracranial pressure) at different cover thicknesses. (**B**) Fourier transform of the pressure wave signal inside the injury apparatus at different cover thicknesses. (**C**) The mean time between the peaks of the pressure wave signal inside the injury apparatus at different cover thicknesses. (**D**) The average wave amplitude of the pressure wave signal inside the injury apparatus at different cover thicknesses.

We further examined the transmitted pressure profiles inside the blast apparatus across the three different cover thicknesses (Figure 7A). An initial evaluation of the pressure profiles found no significant differences in average pressure, impulse, or peak pressure. The average pressure (n = 5) for 1/8″ (3.125 mm) was 27.6 ± 2.8 psi, for 3/16″ (4.8 mm) was 22.9 ± 0.46 psi, and for 1/4″ (6.35 mm) was 24.3 ± 0.9 psi. The pressure impulse (n = 5) for 1/8″ (3.125 mm) was 29.74 ± 1.12 psi·ms, for 3/16″ (4.8 mm) was 31.25± 3.08 psi·ms, and for 1/4″ (6.35 mm) was 32.94± 3.01 psi·ms. The peak pressure (n = 5) for 1/8″ (3.125 mm) was 25.6 ± 3.6 psi, for 3/16″ (4.8 mm) was 24.4 ± 2.1 psi, and for 1/4″ (6.35 mm) was 23.9 ± 1.6 psi. It is worth noting that the standard deviation of the peak pressure decreased as the thickness cover increased; the standard deviation decreased from 3.6 to 2.1 to 1.6 for 1/8″ (3.125 mm), 3/16″ (4.8 mm), and 1/4″ (6.35 mm), respectively, suggesting larger thickness is a more stable system with less variation in peak pressure. This is also evident in viability data, as thicker covers had less variation in neuronal survival, as shown in Figure 3C.

Given that we see a significant difference in viability between the different cover thicknesses, but no difference in analyzed pressure wave parameters, we looked into the dynamics of pressure waveforms. A Fourier transform analysis of the pressure profile revealed higher frequencies were present for the 1/8″ (3.125 mm) cover than for the 3/16″ (4.8 mm) and 1/4″ (6.35 mm) covers (Figure 7B). To confirm the higher frequencies for the thinner covers, we calculated the mean time between the peaks for each pressure signal, which were 6.9 × 10^−5^ ± 0.25 × 10^−5^ s for 1/8″ (3.125 mm), 7.9 × 10^−5^ ± 0.08 × 10^−5^ s for 3/16″ (4.8 mm), and 10.3 × 10^−5^ ± 1.5 × 10^−5^ s for 1/4″ (6.35 mm). The mean time between peaks was significantly different between the 1/8″ (3.125 mm) and 3/16″ (4.8 mm) (F(5) = 28.9, *p* < 0.05) covers and between the 1/8” (3.125 mm) and 1/4″ (4.8 mm) covers (F(5) = 10.09, *p* < 0.05) (Figure 7C).

Finally, the average pressure wave amplitude was calculated, which is the arithmetic average of the deviations of the absolute values of the peak pressure from the mean pressure (Figure 7). The average pressure wave amplitude, equation WA in the Section 2.4, for 1/8″ (3.125 mm) was 5.38 ± 0.2 psi, for 3/16″ (4.8 mm) was 6.22 ± 0.62 psi, and for 1/4″ (6.35 mm) was 6.9 ± 0.5 psi. The average pressure wave amplitude was significantly different between the 1/8″ (3.125 mm) and 1/4″ (4.8 mm) covers (F(5) = 15.66, *p* < 0.05) (Figure 7D).

## 4. Discussion

The overall goal of this study was to replicate the CSF and skull thickness barriers in human anatomy and study their effect on the cell viability of neuronal cultures exposed to a blast wave produced by a laboratory shock tube. This study demonstrated that in vitro neuronal cultures can be successfully adapted to shock waves in air exposure and used to address relevant questions on the direct effects of shocks on the neuronal cell. The results of this study revealed that neuronal cell viability correlated with the thicknesses of the acrylic covers (3.125–6.35 mm), which varied within the published range for human skull thicknesses (2–16 mm) [44,45,46,47]. Interestingly, the thicker cover attenuated the pressure wave frequency inside the blast apparatus but had no effect on the peak pressure or impulse. This finding suggests a potential dependency between the pressure wave frequency and cell viability. In addition, our findings suggest a correlation between peak pressure variability and viability variability. Thicker covers tend to be more stable systems, with lower variability in peak pressure and viability, compared with thinner covers.

This study produced successful methods to recreate a realistic model with anatomic features. From experience with previous blast models, cells cultured on hard plastic substrates tend to detach when subjected to blast injury. This is likely due to the shock interacting with a large change in material properties between the plastic substrate and the surrounding liquid media [34,56]. Furthermore, this is an unrealistic material interface that does not exist in the brain. In the current model, we tested a soft PDMS substrate on which cells remained attached after blast exposure. The media surrounding the cultured cells was used as a simulant of cerebrospinal fluid, both having viscosity similar to water [52,53].

The blast apparatus was designed with an acrylic cover representing the human skull. The Young’s modulus of acrylic is 2.76–3.00 GPa, which is within the range of the human cranial bone tensile modulus of 0.45–10.0 GPa [47,48,49,50,51]. The transmitted pressure wave measured inside the apparatus was within the range of published intracranial pressures from both cadaveric blast testing and surrogate head models [37,57,58,59,60]. Our measured intracranial pressure ranged between 23 to 30 psi, which is similar to computational [37] and other surrogate head models [59]. Interestingly, our model showed that the measured intracranial pressure was higher than the incident pressure by a ratio of 1.3 to 1.9, depending on the cover thickness. This increase in peak pressure has also been demonstrated in a cadaveric model, reporting an intracranial pressure of 37 psi for an incident pressure of 15 psi, which is within the range of our reported data [60]. The increase between the incident and internal pressure has been investigated using a computational model with a shock wave to a system, including a plastic cover, water, and endothelial cells [34,59,60]. The model produced blast waveforms similar to our results, as shown in Figure 5C. The authors proposed the effect was caused by the interface between materials with different compressibilities. As the incident shock wave interacts with the plastic cover, the transmitted wave amplitude has to be the sum of the incident wave and a reflected wave (Newton’s third law). When the compressibility is very high in the air and very low in plastic and water, the reflected wave will have an amplitude almost equal to the incident wave, and the transmitted wave could have an amplitude almost twice as big as that of the incident wave [33,34,35].

Unexpectedly, this study revealed that cell viability scales with pressure wave frequency and not peak pressure. Frequency and amplitude analysis are important measures in brain injury due to the viscoelastic properties of the brain [61,62]. Viscoelastic materials, such as brain tissues, behave with both an elastic response (spring) and a damping response (dashpot) that includes dissipation (damping) and dispersion (delay or phase shift) when undergoing deformation [63,64,65]. In particular, the material will behave with increased stiffness with increased frequency or rate. Indeed, it has been shown that strain rate (frequency) affects neural viability in vitro [61,62,65,66]. This finding suggests that the rate of change and the oscillation of the pressure wave are important in neuronal injury under blast.

Incident wave reflections within the shock tube were evident in this model, most likely due to the large surface area of the blast apparatus compared with the cross-sectional surface area of the blast tube. It has been reported that when the specimen occupies less than 20% of the area inside the shock tube, the shock wave structure and the measured pressure profiles on the surface of the specimen are relatively unaffected by reflections [67,68]. One limitation of our model is that the current apparatus design occupies 36% of the shock tube cross-sectional area. Accordingly, fully understanding the effects of the reflected wave on cell viability can’t be determined without control of the wave reflection. However, our data suggest that the reflected incident wave is transmitted through the injury apparatus and its effects can be seen as a second pressure peak in the transmitted wave, as shown in Figure 6A. Other limitations of our model include that it does not model intracranial geometry, brain tissue heterogeneity, meninges, and vasculature. In addition, our model does not model the skull curvature, as it uses a flat surface instead.

In conclusion, this model successfully blasted injured cells without detaching them and caused a significant change in their viability from a single blast. This model allows an adjustable level of bTBI based on the cover thickness, which is an added value not present in other bTBI models. This model highlights the importance of pressure wave frequency as a significant factor in cell viability in bTBIs. For the same peak pressure, cells can survive low-frequency waves even if they have higher amplitude.

## Data Availability

The datasets used and/or analyzed during the currestudy are available from the corresponding author upon reasonable request.
